# Clinical Factors Associated with hrCT-Confirmed Interstitial Lung Disease in Rheumatoid Arthritis: A Retrospective Case–Control Study

**DOI:** 10.3390/jcm15072735

**Published:** 2026-04-04

**Authors:** Oana-Georgiana Dinache, Claudiu C. Popescu, Corina D. Mogoșan, Cătălin Codreanu, Luminița Enache

**Affiliations:** 1 Rheumatology Department, Carol Davila University of Medicine and Farmacy, 37 Dionisie Lupu Street, District 2, 020021 Bucharest, Romania; oana-georgiana.gutoiu@drd.umfcd.ro (O.-G.D.); corina.mogosan@umfcd.ro (C.D.M.); catalin.codreanu@umfcd.ro (C.C.); luminita.enache@umfcd.ro (L.E.); 2 Ion Stoia Clinical Center for Rheumatic Diseases, 5th Thomas Masaryk Street, District 2, 020983 Bucharest, Romania

**Keywords:** rheumatoid arthritis, interstitial lung disease, high-resolution computed tomography, case–control study

## Abstract

**Background/Objectives**: Rheumatoid arthritis-associated interstitial lung disease (RA-ILD) is a major contributor to morbidity and mortality in RA, yet early recognition remains challenging in routine care. The study aimed to identify clinical factors associated with hrCT-confirmed RA-ILD using a CT-verified case–control design. **Methods**: A single-center retrospective case–control study was designed to include RA patients who underwent chest hrCT in routine care. Cases were patients with ILD on index hrCT (*n* = 79) and controls were RA patients with hrCT negative for ILD (*n* = 59). Data were manually abstracted from clinical interview, laboratory testing, RA activity and structural assessment, respiratory examination, pulmonary function tests (PFT), chest radiography, and hrCT. Predictors were extracted from the 12 months preceding the index scan. Univariate comparisons used nonparametric tests or χ^2^, as appropriate. Prespecified multivariable logistic regression estimated adjusted odds ratios (aORs). Sensitivity analyses included restriction to patients with available pre-index PFT, addition of respiratory examination variables, and a matched conditional logistic regression analysis. **Results**: In the primary multivariable model, male sex was independently associated with RA-ILD (aOR 5.31, 95% CI 1.91–14.75), and COPD/asthma was also associated (aOR 2.82, 1.05–7.56). Adding dyspnea and Velcro crackles improved discrimination (AUC 0.797 to 0.850); Velcro crackles were independently associated with RA-ILD (aOR 5.11, 1.32–19.73). Findings were directionally similar in sensitivity analyses, though precision decreased in matched models. **Conclusions**: In this CT-imaged real-world RA cohort, male sex, COPD/asthma, and Velcro crackles were associated with hrCT-confirmed RA-ILD; these findings should be interpreted as preliminary, as they apply to patients selected for imaging and should not be extrapolated to unselected RA populations without validation in larger, multi-center and/or prospective cohorts with systematic ascertainment.

## 1. Introduction

Interstitial lung disease (ILD) is among the most consequential extra-articular manifestations of rheumatoid arthritis (RA), contributing substantially to morbidity, premature mortality, and medical costs [1,2,3,4]. RA patients have a markedly higher lifetime risk of developing ILD than non-RA comparators and RA-ILD is associated with substantially worse survival [2]. Reported RA-ILD frequency varies widely by geography and, importantly, by ascertainment method: a 2025 meta-analysis estimated a global pooled prevalence of 21% with high heterogeneity [5], whereas systematic hrCT in early RA identified ILD in 11% [6], and routine-care national studies have reported lower frequencies (e.g., 2% of incident RA in Denmark [3] and 5% among RA patients in Spain [7]).

ILD can occur at any stage of the course of RA, including prior to the onset of joint manifestations or shortly after the diagnosis of early RA [8,9,10]. Importantly, systematic imaging suggests that clinically silent or minimally symptomatic ILD is not rare, and protocolized screening of early RA has identified ILD on baseline hrCT in approximately 11% of patients (SAIL-RA), underscoring that reliance on symptoms alone may miss early disease [6]. There are plausible pathogenic mechanisms that support the involvement of mucosal inflammation, especially in the lungs, in the development of RA [11]. Environmental factors, such as smoking or exposure to various inhalers, together with systemic and vascular inflammation, can act synergistically, causing alveolar inflammation and interstitial fibrosis [12]. A current etiopathogenic hypothesis suggests that in genetically predisposed individuals, external factors, especially smoking, induce aberrant citrullination of peptides and alveolar proteins. This phenomenon causes the activation of T and B lymphocytes, followed by the synthesis of anti-citrullinated protein antibodies (ACPA) and the formation of immune complexes, mechanisms that ultimately lead to peripheral arthritis. Consequently, the lung may be involved in RA initially as an extra-articular site of disease initiation, and subsequently as a site of extra-articular manifestation during the course of the disease already constituted [12]. Beyond serology and exposures, genetic susceptibility also contributes; the MUC5B promoter variant (rs35705950) has been associated with RA-ILD, particularly with UIP-pattern disease on imaging, supporting a distinct fibrotic risk architecture in a subset of patients [13]. Contemporary concepts emphasize that clinically overt RA-ILD represents only part of the spectrum, while subclinical parenchymal abnormalities and airway disease are frequently detectable with high-resolution computed tomography (hrCT) and pulmonary function testing (PFT), particularly when systematic assessment is pursued [14].

A persistent challenge is that RA-ILD is often under-recognized early, because symptoms such as exertional dyspnea or cough are non-specific and may be attributed to comorbidity or deconditioning. Several screening strategies for the detection of RA-ILD have been proposed, based on known epidemiological risk factors. The 2023 guidelines of the American College of Rheumatology (ACR) and the American College of Chest Physicians (CHEST), recommend screening asymptomatic patients diagnosed with autoimmune rheumatic conditions, including RA, who have risk factors for lung damage [15]. Risk factors identified in the development of lung damage in RA patients include increased titers of rheumatoid factor (RF) and ACPA, smoking (active or historical), old age at RA onset (above 60 years), increased RA activity, male sex and increased body mass index, as well as the presence of the MUC5B gene, recognized as a major genetic susceptibility factor for RA ILD [13,15,16]. However, many observational syntheses emphasize substantial heterogeneity across cohorts and case definitions, and the strength of association for individual predictors varies depending on whether ILD is defined clinically, by HRCT screening, or by incident outcomes [5,17,18,19]. Other studies propose weighted risk scores that integrate combinations of demographic and lifestyle factors, along with determinations of autoantibodies and/or inflammatory markers [20,21,22]. In addition, the ongoing ANCHOR-RA study [23], which investigates the prevalence of RA-associated ILD in high-risk populations, selected five recognized epidemiological risk factors as inclusion criteria. Notably, in early RA populations undergoing systematic HRCT, several proposed screening strategies show variable sensitivity/specificity trade-offs, suggesting that transportability and calibration across settings remain open questions. However, the performance of these screening strategies in independent populations, especially in patients with newly diagnosed RA, remains insufficiently clarified.

Given these constraints, pragmatic approaches that enrich pre-test probability and trigger timely evaluation are attractive. In fibrotic ILD more broadly, “Velcro-type” inspiratory crackles on auscultation correlate with fibrotic changes on hrCT and may support earlier identification [24]. In RA, the feasibility of systematizing this bedside signal has been reinforced by validation of digital crackle detection (VECTOR), which demonstrated clinically meaningful diagnostic accuracy against hrCT in a multi-center RA cohort [25]. However, in real-world practice, hrCT is typically performed selectively (e.g., for symptoms, abnormal clinical examination, or abnormal chest radiography), which can introduce verification/selection effects and complicate inference regarding risk factors versus associations among imaged patients. Accordingly, studies using hrCT-negative controls can reduce misclassification of non-imaged patients as ILD negative, but still require cautious interpretation because the imaged population is not equivalent to an unselected RA population [26,27].

In this context, we hypothesized that, among RA patients undergoing chest hrCT in routine care, hrCT-confirmed RA-ILD would be independently associated with a high-risk clinical phenotype beyond RA disease duration and treatment exposures. Accordingly, the purpose of this study was to identify clinical factors independently associated with hrCT-confirmed RA-ILD by comparing RA patients with hrCT-proven ILD to RA patients with hrCT negative for ILD in a single-center, retrospective case–control design using structured data abstraction across clinical, laboratory, RA activity/imaging, respiratory examination, pulmonary function, and hrCT domains.

## 2. Materials and Methods

### 2.1. Study Design, Setting and Patients

This single-center, retrospective case–control study used routinely collected electronic medical record (EMR) data from a tertiary rheumatology service with integrated access to radiology and pulmonary function testing (PFT), between February 2025 and January 2026. All adult patients with a clinical diagnosis of RA, fulfilling the 2010 classification criteria [28], who had at least one chest high-resolution computed tomography (hrCT) performed within the study period were eligible for screening. Patients were included if sufficient information was available to ascertain RA status, the hrCT result with respect to ILD, and key covariates within the prespecified look-back window. For patients with multiple CT examinations, the earliest qualifying scan according to the group definition (see below) was considered the index scan. Cases were defined as RA patients with ILD identified on hrCT, with the index date defined as the date of the first ILD-positive hrCT. Controls were defined as RA patients with a chest hrCT negative for ILD, with the index date defined as the date of the ILD-negative hrCT. Where applicable, ILD patterns on hrCT were recorded from radiology reports and categorized into usual interstitial pneumonia (UIP), nonspecific interstitial pneumonia (NSIP), or other/unclassifiable patterns. For each participant, clinical predictors and covariates were abstracted using a fixed pre-index look-back window defined as 365 days before the index date through 1 day before the index date (index-365 to index-1). When multiple measurements were available within the look-back window, the value closest to the index date was preferentially recorded. Although the prespecified abstraction window was 365 days before the index hrCT, for dynamic variables (inflammatory markers, RA activity, and respiratory assessment) the measurement/assessment closest to the index hrCT date was extracted rather than summarizing values across the full year.

The study used retrospective, routinely collected clinical data. Data were de-identified prior to analysis and handled according to institutional data protection policies. All patients gave informed consent for scientific use of data and the study was approved by the local ethics committee.

### 2.2. Data Sources and Extraction Procedure

Data were extracted manually from the EMR using a standardized data collection workbook. Source documents included structured EMR fields (demographics, diagnoses, medication lists), rheumatology clinic notes (history, physical examination, disease activity), laboratory results, imaging reports, PFT reports, and chest radiography, and hrCT reports. To improve reproducibility, variable definitions and extraction rules were prespecified in a data dictionary. Data abstraction focused on the pre-index period to ensure temporality (i.e., predictors preceded or coincided with the index imaging event rather than being consequences of it).

Clinical interview data were abstracted from rheumatology visits within the look-back window and included demographics (age at index, sex), smoking history (never/former/current and pack-years when available), respiratory symptoms (dyspnea and chronic cough), and relevant comorbidities (e.g., COPD/asthma, cardiovascular disease, diabetes mellitus, chronic kidney disease, gastroesophageal reflux if available). When multiple visit notes were present, symptom presence and severity were recorded as the worst documented status during the pre-index window.

Laboratory variables were retrieved from the institutional laboratory system and included inflammatory markers (C-reactive protein [CRP], erythrocyte sedimentation rate [ESR]) and RA serology (rheumatoid factor [RF] and anti-citrullinated protein antibody [ACPA] status and titers when available). For CRP and ESR, the value closest to the index date within the look-back window was recorded. For RF and ACPA, the most recent result prior to index (or closest available) was used. RA disease duration was calculated from the documented date of RA diagnosis (or earliest definitive RA documentation) to the index date. RA activity was captured using routinely documented composite indices (DAS28-CRP where available). Structural disease severity was recorded from available rheumatology imaging assessments (radiographic stage and erosive status) as documented in the EMR or imaging summaries.

Medication exposure variables were abstracted from prescription records and medication lists and included exposure to methotrexate and leflunomide (categorized as current, former, or never), current biologic/targeted synthetic disease-modifying anti-rheumatic drug (b/tsDMARD) class, and current systemic glucocorticoid use (yes/no and dose category where available). Medication status was assigned relative to the index date using the most contemporaneous medication reconciliation and prescription history. To minimize bias from treatment discontinuation triggered by respiratory symptoms, the primary regression analyses used ever exposure (current or former versus never) for methotrexate and leflunomide.

Respiratory examination findings were abstracted from rheumatology and pulmonary examinations documented during the look-back window. Key findings included the presence of Velcro-type inspiratory crackles and resting oxygen saturation (when available). Velcro crackles were coded as present when explicitly described and coded as absent when the examination explicitly reported normal auscultation or absence of crackles. When multiple examinations were recorded, findings were coded as present if documented at least once within the pre-index window. PFT availability prior to index was recorded (yes/no). When available, spirometric indices (forced vital capacity [FVC] % predicted; forced expiratory volume in 1 s [FEV1] % predicted) and diffusing capacity (DLCO % predicted) were abstracted from PFT reports, selecting the test closest to the index date within the look-back window. Chest radiography performed within the 12 months preceding index was recorded (yes/no). Where applicable, chest X-ray reports were reviewed for descriptors compatible with ILD and coded as chest X-ray suggestive of ILD (yes/no) based on radiology reporting language. Index chest hrCT dates were recorded for all subjects. For cases, chest hrCT findings were abstracted from the original radiology reports issued in routine clinical care. Cases were defined by the presence of ILD-compatible changes on hrCT, and controls by an index hrCT explicitly reported as negative for ILD. HRCT pattern (UIP/NSIP/other/unclassifiable) was recorded when specified but was used for descriptive characterization rather than to define case status. No centralized blinded re-reading by an ILD-specialist radiologist was performed. Additional CT findings relevant to pulmonary comorbidity and differential diagnosis were recorded when reported, including bronchiectasis, emphysema, and rheumatoid nodules.

### 2.3. Statistics

Continuous variables were assessed for distributional normality using the Shapiro–Wilk test (Supplementary Table S1) within each group and are presented as mean ± standard deviation (SD) when approximately normally distributed in both groups, otherwise as median [first quartile, third quartile]. Categorical variables are reported as n (%), using non-missing denominators. Between-group comparisons used Welch’s *t*-test for normally distributed continuous variables and the Mann–Whitney U (or Kruskal–Wallis where applicable) test for non-normally distributed variables. Categorical variables were compared using χ^2^ tests, with Fisher’s exact test applied for cross tables when expected cell counts were <5.

An unmatched retrospective case–control analysis was performed to identify clinical factors associated with hrCT-confirmed RA-associated ILD. Cases were RA patients with ILD on index hrCT, and controls were RA patients with hrCT negative for ILD at the index scan date. The index date was defined as the first ILD-positive hrCT for cases and the hrCT date for controls. Candidate predictors were pre-specified based on clinical plausibility and included demographics (age, sex), smoking status, RA disease duration, serostatus (RF and ACPA positivity), respiratory comorbidity (COPD/asthma), and treatment exposures before index (ever methotrexate, ever leflunomide, and current systemic glucocorticoids). Multivariable logistic regression was used to estimate adjusted odds ratios (aORs) with 95% confidence intervals (CI). Analyses used complete-case data for model covariates. Sensitivity analyses included repeating the primary model restricted to patients with available pre-index PFTs and an exploratory model additionally adjusting for pre-index respiratory findings (dyspnea and Velcro crackles), acknowledging their potential role as ascertainment-related variables. As an additional sensitivity analysis, we constructed a matched case–control sample to evaluate the robustness of the primary associations. Each RA-ILD case was matched to up to two hrCT-negative controls using a greedy nearest-neighbor algorithm without replacement, requiring exact matching on sex, age within ±5 years, and calendar year of the index CT within ±2 years. Associations were then re-estimated using conditional logistic regression stratified by matched set, which accounts for the matched design and implicitly adjusts for the matching factors. Smoking status was modeled as a categorical covariate (never/former/current), and the remaining covariates mirrored the primary prespecified model. Only matched subjects with complete data for model covariates were included in the conditional regression.

All tests were two-sided with α = 0.05. Univariate *p*-values were reported for descriptive purposes and were not adjusted for multiple comparisons; inference focused on pre-specified multivariable models. All analyses were performed using IBM SPSS Statistics (version 26.0; IBM, Armonk, NY, USA) and Python (version 3.4).

## 3. Results

### 3.1. Univariate Analysis

In this retrospective case–control sample of 138 RA patients (79 RA-ILD cases and 59 CT-negative controls), mean age was similar between groups’ (67.0 [61.5, 71.7] years in cases versus 66.0 [57.4, 71.0] years in controls; *p* = 0.150). Sex distribution differed, with a higher proportion of men among cases (43.0% versus 18.6%; *p* = 0.006). Smoking status also differed (*p* = 0.002), with cases showing more current smokers, whereas controls had more former smokers. CRP/ESR/DAS28 and respiratory assessments were largely contemporaneous with imaging: 111/138 (80.4%) were recorded on the same date as the index hrCT, 119/138 (86.2%) within 30 days, and 137/138 (99.3%) within 90 days of the index scan. Same-day documentation occurred in 57/79 (72.2%) of cases and 54/59 (91.5%) of controls, while 78/79 (98.7%) of cases and 59/59 (100%) of controls had these assessments within 90 days.

RA duration, serostatus, inflammatory markers (CRP, ESR), DAS28-CRP, radiographic stage, erosive status, csDMARD exposure (methotrexate, leflunomide), b/tsDMARD class, and current glucocorticoid use did not differ significantly (all *p* > 0.05; Table 1). Respiratory manifestations were documented for all included participants within the pre-index window and were more frequent in cases, including dyspnea (38.0% versus 15.3%; *p* = 0.006) and Velcro crackles (39.2% versus 6.8%; *p* < 0.001), while chronic cough showed a nonsignificant trend (35.4% versus 20.3%; *p* = 0.081). On index CT, bronchiectasis was more common in cases (36.7% versus 18.6%; *p* = 0.034), while rheumatoid nodules and emphysema did not differ. Pre-index chest radiography suggestive of ILD was markedly more frequent in cases (57.0% versus 25.4%; *p* < 0.001). Among patients with available pre-index PFTs, FVC was lower in cases (92.1 ± 22.3% versus 101.3 ± 22.9%; *p* = 0.042), whereas FEV1 and DLCO did not differ significantly.

### 3.2. Multivariate Analysis

**Figure 1:**
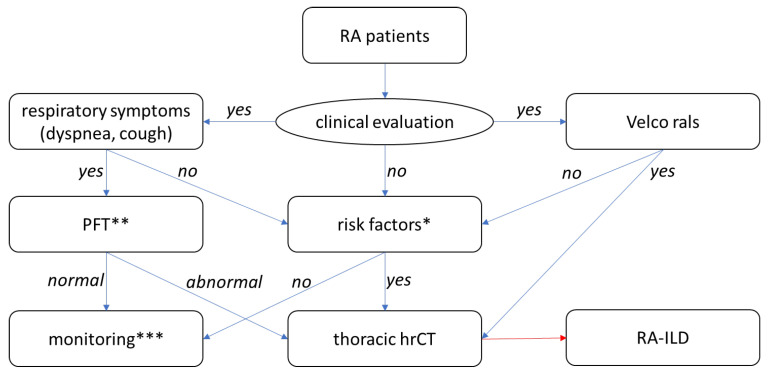
Conceptual (hypothesis-generating) schematic for consideration of hrCT evaluation in RA. * Risk factors: age over 60 years at RA diagnosis, male sex, smoking (current or historical), long disease duration, moderate-high RA activity, positive RF and/or ACPA. ** PFT: spirometry, DLCO. *** Monitoring every 6 months for respiratory symptoms (especially dyspnea, dry cough), signs (Velcro rales) and lung function (6-min walk test, PFT). Abbreviations: ACPA, anti-citrullinated protein antibodies; DLCO, diffusing capacity of the lungs for carbon monoxide; hrCT, high-resolution computed tomography; ILD, interstitial lung disease; PFT, pulmonary function tests; RA, rheumatoid arthritis; RF, rheumatoid factor.

In the unmatched case–control analysis (Table 2), 134/138 patients had complete data for the prespecified covariates (77 cases and 57 controls overall). In the primary multivariable logistic regression model, male sex was independently associated with higher odds of RA-ILD (aOR 5.31, 95% CI 1.91–14.75; *p* = 0.001). Current smoking (versus never) was associated with higher odds of RA-ILD (aOR 3.05, 95% CI 0.83–11.24; *p* = 0.094), although non-significant, while former smoking was associated with lower odds of RA-ILD (aOR 0.28, 95% CI 0.10–0.81; *p* = 0.019). COPD/asthma was associated with higher odds of RA-ILD (aOR 2.82, 95% CI 1.05–7.56; *p* = 0.040). RF positivity also showed a borderline association (aOR 3.44, 95% CI 0.98–12.11; *p* = 0.054). Age, RA duration, ACPA positivity, ever methotrexate exposure, ever leflunomide exposure, and current glucocorticoid use were not independently associated (all *p* > 0.05). Model discrimination was good (AUC 0.797) with McFadden pseudo-R2 of 0.211. Based on these results and existing guideline-based approaches, we provide a hypothetical, conceptual algorithm to illustrate how bedside findings and established risk factors could be integrated to prompt hrCT consideration in routine care (Figure 1).

### 3.3. Sensitivity Analyses

In sensitivity analysis restricted to patients with available pre-index PFTs (*n* = 96; Table 3), male sex remained significant (aOR 5.57, 95% CI 1.49–20.84; *p* = 0.011), and RF positivity became statistically significant but imprecise (aOR 12.82, 95% CI 1.27–129.50; *p* = 0.031).

An exploratory model additionally adjusting for pre-index dyspnea and Velcro crackles (Table 4) was performed to examine whether respiratory findings documented in routine care were associated with hrCT-confirmed RA-ILD among CT-imaged patients. In this model, Velcro crackles were independently associated with RA-ILD (aOR 5.11, 95% CI 1.32–19.73; *p* = 0.018), while male sex remained significant and current smoking remained non-significant. As an additional sensitivity analysis (Table 5), age-, sex-, and CT-year-matched conditional logistic regression (29 matched sets; 28 cases/48 controls after complete-case restriction) yielded directionally similar results but with wide confidence intervals; COPD/asthma remained associated with RA-ILD (aOR 39.12, 95% CI (1.46–1045.61; *p* = 0.029), while current smoking was non-significance (*p* = 0.876).

The repeated primary multivariable model using a narrower 90-day pre-index window (index-90 to index-1) for CRP, ESR, DAS28, and respiratory variables revealed that results were unchanged and only one subject was excluded due to lack of measurements within 90 days. In addition, the measurement-to-CT interval (days) was included as a covariate in models incorporating respiratory variables; the interval was not independently associated with case status and did not materially alter effect estimates for the main associations.

The comparison of baseline characteristics between participants included in the primary multivariable model and those excluded due to missing covariates, and participants included in the matched conditional model versus those not retained after matching/complete-case restriction (Supplementary Tables S2 and S3), showed that differences were small and did not suggest major systematic bias.

## 4. Discussion

RA-ILD frequency and outcomes vary across geographic regions and racial/ethnic groups, reflecting both biologic susceptibility and differences in ascertainment (systematic hrCT screening versus clinically detected ILD), referral patterns, and access to specialized care. Global meta-analyses report wide heterogeneity and region-to-region variation in estimated RA-ILD prevalence [5], consistent with major differences in study design and diagnostic pathways. In US administrative analyses [29], race/ethnicity has been associated with differences in ILD-related outcomes among patients with RA, suggesting that the contribution of ILD-to-disease burden may not be uniform across populations. Genetic architecture may further contribute to geographic variability: the MUC5B rs35705950 promoter variant, which is strongly associated with RA-ILD, particularly UIP-pattern disease in European-ancestry cohorts, has markedly different background frequencies by ancestry, affecting population-attributable risk and transportability of risk models [13,30,31]. Thus, the effect estimates from single-center, CT-imaged cohorts should be interpreted cautiously and validated studies that include diverse populations are needed.

### 4.1. Male Sex as a Robust RA-ILD Correlate

In our hrCT-verified case–control cohort, male sex emerged as the most consistent independent correlate of RA-ILD, remaining significant in the primary multivariable model and across sensitivity analyses. This aligns with a large body of RA-ILD literature in which male sex repeatedly appears among the strongest demographic risk markers for ILD development in RA. Recent meta-analyses aggregating observational cohorts identify male sex as a reproducible factor associated with RA-ILD, typically with an OR of up to 2 [17] across heterogeneous case definitions and settings [32]. In population-based longitudinal data, men with RA also show higher incident ILD risk than women [2], supporting that this is not merely a clinic referral artifact. Several, not mutually exclusive, explanations are plausible. First, smoking intensity and cumulative exposure historically differ by sex in many cohorts, and smoking is a well-established RA-ILD risk factor; classic work has suggested that smoking may partly explain the higher frequency of RA-ILD observed in men (particularly via seropositive disease biology) [33]. Second, sex-linked immunobiology and fibrotic susceptibility could contribute: male sex has been repeatedly associated with fibrotic ILD phenotypes and worse ILD outcomes, and RA-ILD reviews consistently list age and male sex among the most frequently reported risk factors [34,35]. Third, male sex may capture a composite of exposure history, comorbidity burden (e.g., airway disease), and diagnostic timing, such that men present later and with more established lung involvement; this is compatible with the persistence of the association even after adjustment for smoking status in many datasets [36,37].

### 4.2. RF and ACPA as Correlates of RA-ILD

Across published RA-ILD cohorts, RF seropositivity, especially higher RF titers, tend to associate with RA-ILD presence and/or risk, although the strength of association varies by design, case definition (prevalent versus incident ILD), and adjustment set. Recent meta-analyses of observational studies usually identify RF positivity among the recurrent risk factors for RA-ILD (alongside age, male sex, smoking, pulmonary comorbidity, and disease activity), but they also highlight heterogeneity and variable evidence quality [17,32]. RF reflects seropositive RA biology ant it often correlates with more severe systemic/extra-articular disease, and may track immune pathways linked to pulmonary involvement. Importantly, multiple datasets suggest that RF titer carries more information than RF “positive/negative”. In a large cohort analysis evaluating autoantibodies and RA-ILD, RF and ACPA were associated with prevalent RA-ILD, but for incident RA-ILD, the association emerged mainly at higher RF concentrations [33], consistent with a threshold effect rather than a linear relationship. More recent work similarly reports nonlinear/threshold patterns between RF and RA-ILD risk [34]. Older studies also found that high RF titers (not merely positivity) are associated with increased ILD risk within RA populations [35].

In our cohort, RF positivity showed a plausible but statistically unstable association with RA-ILD across models. In the prespecified primary multivariable model, RF positivity was marginally non-significant (aOR 3.44, 95% CI 0.98–12.11, *p* = 0.054), but became significant in the PFT analysis, but became non-significant after adding dyspnea/Velcro crackles and in the matched analysis, suggesting that any RF signal was not solely explained by symptom-driven ascertainment. In the PFT-restricted sensitivity analysis, RF positivity became statistically significant but highly imprecise, consistent with a scenario where restricting to patients with more complete pulmonary assessment changes the case–control mix and amplifies sparse-data effects. In the matched conditional model, RF produced a non-significant and extreme estimate (aOR 2.51, 0.90–54.22, *p* = 0.968), reflecting quasi-separation in a small matched subset and reinforcing that RF inference is fragile when sample size is reduced. This overall pattern is biologically and epidemiologically plausible, since RF has repeatedly been linked to RA-ILD in meta-analyses and risk-factor syntheses, but it also highlights an important nuance: in large longitudinal data, RF “titer” (especially high concentrations) may be more informative than a simple positive/negative status, with incident RA-ILD risk increasing primarily above higher RF thresholds rather than across all RF-positive patients [33]. The behavior of RF as an ILD predictor in our study supports it as a candidate marker of RA-ILD susceptibility, but it also indicates that, in a retrospective hrCT-selected case–control design, its independent effect is sensitive to sampling and modeling choices and should be interpreted as suggestive unless confirmed using RF titers/thresholds in larger, ideally prospective, cohorts.

Although high-titer ACPA has been associated with RA-ILD in multiple cohorts and may interact with genetic susceptibility (e.g., MUC5B) [13], ACPA positivity was not positively associated with RA-ILD in our CT-verified case–control sample. We caution against interpreting this as a protective effect. Our ACPA measure was primarily binary (positive/negative), which may obscure the titer/threshold effects reported in the literature. Additionally, our control group comprised hrCT-imaged RA patients negative for ILD rather than unselected RA patients; such a design may enrich controls for respiratory evaluation and seropositive disease, attenuating associations and increasing collinearity with RF and smoking. The wide confidence intervals further suggest limited precision.

### 4.3. Velcro Crackles Predict RA-ILD

In our cohort, Velcro-type inspiratory crackles emerged as a high-yield clinical sign for RA-ILD: when added to the prespecified multivariable model, crackles were independently associated with hrCT-confirmed RA-ILD (aOR 5.17, 95% CI 1.34–19.92; *p* = 0.017) and improved discrimination (AUC increased from 0.797 in the primary model to 0.850 in the sensitivity analysis model). However, Velcro-type crackles should be interpreted as a pragmatic case-finding cue in routine rheumatology practice: they are likely to reflect underlying fibrotic parenchymal involvement, but they may also influence the probability of undergoing hrCT. Accordingly, our findings support their clinical usefulness for enriching pre-test probability among evaluated patients, while prospective studies with systematic hrCT are needed to quantify their independent screening performance and avoid ascertainment bias.

This finding is consistent with a broader fibrotic ILD literature showing that Velcro-type crackles predict the presence of fibrotic ILD and correlate with radiologic extent, providing a plausible pathophysiologic link between auscultatory findings and parenchymal fibrosis burden [24,36]. In RA specifically, crackles are frequently reported in established RA-ILD, but can also occur in RA without ILD, emphasizing that they function best as a screening prompt rather than a diagnostic endpoint [37]. Importantly, the growing interest in digitized auscultation supports feasibility for systematic case finding: the VECTOR electronic sound detector has been validated against hrCT in RA, reporting high diagnostic accuracy for ILD detection, which aligns with our observation that “traditional” clinical examination captures meaningful signal even before incorporating more advanced tools [25]. Careful respiratory examination for the detection of Velcro crackles can meaningfully enrich pre-test probability and should lower the threshold for PFTs and/or hrCT in RA patients, while acknowledging that crackles may also reflect real-world referral pathways (i.e., clinicians are more likely to order hrCT when crackles are present).

### 4.4. Practical Information

From a practical standpoint, our findings can be translated into a simple, clinic-oriented message: RA patients who are male and/or have COPD/asthma deserve a lower threshold for pulmonary evaluation, and Velcro-type inspiratory crackles should prompt expedited work-up. In routine rheumatology visits, physicians should actively ask about exertional dyspnea and dry cough, perform careful lung auscultation, and review prior chest radiography. When crackles are present or when a high-risk patient (male, COPD/asthma, seropositive RA, smoking) reports new respiratory symptoms, clinicians should obtain PFTs including DLCO and consider hrCT (or pulmonology referral) even if symptoms are mild, as bedside findings significantly enrich the probability of hrCT-detectable ILD in CT-imaged cohorts. Conversely, because this study is restricted to CT-imaged patients, these factors should be viewed as case-finding cues rather than a validated screening rule.

### 4.5. Limitations and Further Studies

This study has several limitations inherent to its design and data source. The results should be interpreted in the context of a CT-selected sample: inclusion required chest hrCT, so associations reflect correlates of ILD among RA patients referred for imaging, not population-level risk factors. It is a single-center, retrospective chart-review study, which limits generalizability and introduces potential information bias from incomplete or inconsistent EMR documentation. Second, because the analytic sample was restricted to RA patients who underwent chest hrCT, the study is subject to selection/verification bias: the results primarily address correlates of RA-ILD among CT-imaged RA patients, and cannot be directly extrapolated to all RA patients who never underwent CT. Several predictors were measured within a pragmatic pre-index window and may be affected by time-dependent confounding or reverse causation (e.g., medication changes or smoking cessation after onset of respiratory symptoms). Although auscultation was documented for all included patients, respiratory examination was performed as part of routine clinical practice and was not standardized across clinicians; thus, inter-observer variability and differences in documentation granularity may persist, and the association observed for Velcro crackles should be interpreted as reflecting real-world practice rather than a protocolized diagnostic accuracy estimate. Because hrCT was performed in routine care rather than systematically, respiratory findings (including crackles) may have influenced imaging decisions, introducing ascertainment bias. We therefore treat crackles as an exploratory real-world marker among CT-imaged patients, not as a causal determinant of ILD or a validated screening test. Methotrexate and leflunomide are time-varying exposures that may be discontinued when ILD is suspected; therefore, residual protopathic bias cannot be fully excluded in a retrospective design. Treatment exposures were captured as current/former/never (or “ever”) rather than cumulative dose or duration, and confounding by indication cannot be excluded; therefore, medication-related findings should not be interpreted causally. The matched sensitivity analysis was limited by small sample size, resulting in imprecise estimates and occasional sparse-data phenomena. This reflects limited information per parameter and potential collinearity in retrospective data. Accordingly, these estimates are hypothesis generating and require confirmation in larger, preferably multi-center, cohorts. Complete-case modeling may also have introduced informative missingness if missingness was related to disease severity or work-up intensity (e.g., PFT availability). ILD characterization relied primarily on radiology reports rather than centralized multidisciplinary adjudication and did not include quantitative hrCT scoring; thus, phenotype misclassification (pattern assignment, extent) is possible. Inter-reader variability, differences in scanner protocols, and heterogeneity in expertise may have resulted in misclassification, particularly for ILD pattern subtyping; this limitation may attenuate associations and limits inferences regarding UIP/NSIP distribution. In patients with COPD, hrCT abnormalities may mimic or coexist with ILD, and reliance on routine reports without centralized re-reading may lead to misclassification, particularly in mixed airway–interstitial phenotypes. Such misclassification could bias the observed COPD association in either direction and limits causal interpretation.

Future research should validate these findings in larger, multi-center cohorts with standardized capture of smoking exposure (pack-years and cessation timing), symptoms, and auscultation. The proposed schematic in Figure 1 requires external validation (prospective cohorts with systematic outcome ascertainment) before any clinical use. Prospective designs using prespecified screening pathways would better address temporality and reduce CT-selection bias. Incorporating longitudinal PFTs, quantitative hrCT scoring, standardized hrCT acquisition protocols, and ideally multidisciplinary ILD adjudication would improve phenotyping and allow analyses by ILD subtype and severity. Future studies should model RF and ACPA as titer and incorporate genetic susceptibility and ILD subtype to clarify the independent role of ACPA in RA-ILD risk. Finally, any multivariable model should be externally validated before being proposed for clinical risk stratification or screening.

## 5. Conclusions

In this retrospective hrCT-verified case–control study, RA-ILD was independently associated with male sex and coexisting COPD/asthma, with marginal signals for RF positivity and current smoking, and was strongly linked to abnormal respiratory findings, particularly Velcro crackles, consistent with their value as an associated finding among CT-imaged RA patients. By contrast, RA duration, ACPA positivity, and commonly used RA treatments (methotrexate, leflunomide, and glucocorticoids) were not independently associated with RA-ILD in adjusted analyses. These findings support targeted vigilance for RA-ILD in RF-positive smoking male patients and those with pulmonary comorbidity or abnormal lung examination.

## Figures and Tables

**Table 1:** Baseline characteristics of RA patients with hrCT-confirmed ILD and hrCT-negative controls.

Variable	Cases (*n* = 79)	Controls (*n* = 59)	*p*
Age, years	67.0 [61.5, 71.7]	66.0 [57.4, 71.0]	0.150
Female sex	45 (57.0%)	47 (79.7%)	0.006
RA duration, years	7.7 [1.0, 13.0]	7.9 [1.8, 14.0]	0.595
Smoking exposure, pack-years	0.0 [0.0, 30.0]	0.0 [0.0, 8.0]	0.296
RF positive	67 (84.8%)	42 (71.2%)	0.083
ACPA positive	61 (77.2%)	47 (79.7%)	0.892
CRP, mg/L	10.2 [4.1, 25.1]	8.1 [2.1, 20.9]	0.255
ESR, mm/h	38.0 [29.0, 59.5]	27.0 [14.0, 64.5]	0.104
DAS28-CRP	3.7 [2.8, 5.4]	4.1 [2.8, 5.1]	0.987
FVC, % predicted	92.1 ± 22.3	101.3 ± 22.9	0.077
FEV1, % predicted	83.4 ± 23.9	86.6 ± 26.4	0.588
DLCO, % predicted	62.1 ± 25.1	67.9 ± 21.3	0.253
Current smokers	23 (28.6%)	4 (6.9%)	0.002
Former smokers	18 (23.4%)	24 (41.4%)	0.002
Never smokers	36 (46.8%)	30 (51.7%)	0.002
Radiographic stage 1	12 (15.2%)	11 (18.6%)	0.117
Radiographic stage 2	43 (54.4%)	23 (39.0%)	0.117
Radiographic stage 3	16 (20.3%)	11 (18.6%)	0.117
Radiographic stage 4	8 (10.1%)	14 (23.7%)	0.117
Erosions present	67 (84.8%)	48 (81.4%)	0.758
Current methotrexate	30 (38.0%)	23 (39.0%)	0.397
Former methotrexate exposure	38 (48.1%)	32 (54.2%)	0.397
Never exposed to methotrexate	11 (13.9%)	4 (6.8%)	0.397
Current leflunomide	16 (20.3%)	19 (32.2%)	0.219
Former leflunomide	36 (45.6%)	20 (33.9%)	0.219
Never leflunomide exposure	27 (34.2%)	20 (33.9%)	0.219
Abatacept	7 (8.9%)	2 (3.4%)	0.139
Anti-TNF	10 (12.7%)	9 (15.3%)	0.139
JAK inhibitor	4 (5.1%)	3 (5.1%)	0.139
Rituximab	7 (8.9%)	9 (15.3%)	0.139
Tocilizumab	10 (12.7%)	1 (1.7%)	0.139
No b/tsDMARD	41 (51.9%)	35 (59.3%)	0.139
Current GC	20 (25.3%)	11 (18.6%)	0.470
10 mg/day GC	7 (8.9%)	2 (3.4%)	0.512
15 mg/day GC	0 (0.0%)	1 (1.7%)	0.512
20 mg/day GC	1 (1.3%)	0 (0.0%)	0.512
5 mg/day GC	9 (11.4%)	7 (12.1%)	0.512
5–10 mg/day GC	3 (3.8%)	1 (1.7%)	0.512
Dyspnea (last 12 months)	30 (38.0%)	9 (15.3%)	0.006
Chronic cough (last 12 months)	28 (35.4%)	12 (20.3%)	0.081
Velcro crackles (last 12 months)	31 (39.2%)	4 (6.8%)	<0.001
COPD/asthma	26 (32.9%)	11 (18.6%)	0.093
Cardiovascular disease	65 (82.3%)	47 (79.7%)	0.866
Diabetes mellitus	21 (26.6%)	8 (13.6%)	0.100
Chronic kidney disease	15 (19.0%)	8 (13.6%)	0.538
Chest X-ray (last 12 months)	71 (89.9%)	55 (93.2%)	0.700
Chest X-ray suggestive of ILD	45 (57.0%)	15 (25.4%)	<0.001
PFT available pre-index	59 (74.7%)	40 (67.8%)	0.485
Rheumatoid nodules on index CT	34 (43.0%)	29 (49.2%)	0.589
Bronchiectasis on index CT	29 (36.7%)	11 (18.6%)	0.034
Emphysema on index CT	17 (21.5%)	9 (15.3%)	0.477

Abbreviations: ACPA, anti-citrullinated protein antibody; b/tsDMARD, biologic/targeted synthetic disease-modifying antirheumatic drug; COPD, chronic obstructive pulmonary disease; CRP, C-reactive protein; CT, computed tomography; DAS28, Disease Activity Score in 28 joints; DLCO, diffusing capacity of the lung for carbon monoxide; ESR, erythrocyte sedimentation rate; FEV1, forced expiratory volume in 1 s; FVC, forced vital capacity; GC, glucocorticoids; hrCT, high-resolution computed tomography; ILD, interstitial lung disease; JAK, Janus kinase; PFT, pulmonary function tests; RA, rheumatoid arthritis; RF, rheumatoid factor; TNF, tumor necrosis factor.

**Table 2:** Multivariable logistic regression for factors associated with hrCT-confirmed RA-ILD (primary analysis).

Predictor (Reference)	aOR (95% CI)	*p*
Male sex (ref: female)	5.31 (1.91–14.75)	0.001
Former smoker (ref: never)	0.28 (0.10–0.81)	0.019
Current smoker (ref: never)	3.05 (0.83–11.24)	0.094
Age (per year)	1.04 (0.99–1.09)	0.130
RA duration (per year)	0.98 (0.94–1.03)	0.480
RF positive (ref: negative)	3.44 (0.98–12.11)	0.054
ACPA positive (ref: negative)	0.49 (0.14–1.77)	0.280
COPD/asthma (ref: no)	2.82 (1.05–7.56)	0.040
Ever methotrexate (ref: never)	0.53 (0.12–2.23)	0.384
Ever leflunomide (ref: never)	1.06 (0.45–2.51)	0.889
Current glucocorticoids (ref: no)	1.90 (0.69–5.18)	0.212

Model metrics: *n* = 134 (cases = 77, controls = 57); AUC = 0.797; Brier = 0.181; Log-likelihood = −72.14; AIC = 168.29; BIC = 203.06; McFadden R^2^ = 0.211; Hosmer–Lemeshow χ^2^(8) = 10.63, *p* = 0.224. “Unknown” smoking was excluded from modeling. Abbreviations: ACPA, anti-citrullinated protein antibody; AIC, Akaike information criterion; aOR, adjusted odds ratio; AUC, area under the ROC curve; BIC, Bayesian information criterion; CI, confidence interval; COPD, chronic obstructive pulmonary disease; hrCT, high-resolution computed tomography; ILD, interstitial lung disease; RA, rheumatoid arthritis; RF, rheumatoid factor.

**Table 3:** Sensitivity analysis: primary model restricted to patients with available pre-index PFTs.

Predictor (Reference)	aOR (95% CI)	*p*
Male sex (ref: female)	5.57 (1.49–20.84)	0.011
Former smoker (ref: never)	0.32 (0.08–1.23)	0.097
Current smoker (ref: never)	3.80 (0.76–18.92)	0.103
Age (per year)	1.02 (0.96–1.08)	0.509
RA duration (per year)	1.00 (0.95–1.05)	0.990
RF positive (ref: negative)	12.82 (1.27–129.50)	0.031
ACPA positive (ref: negative)	0.08 (0.01–1.01)	0.051
COPD/asthma (ref: no)	2.39 (0.74–7.72)	0.144
Ever methotrexate (ref: never)	0.39 (0.06–2.62)	0.336
Ever leflunomide (ref: never)	1.22 (0.43–3.42)	0.708
Current glucocorticoids (ref: no)	1.92 (0.53–6.98)	0.323

Model metrics: *n* = 97 (cases = 58, controls = 39); AUC = 0.790; Brier = 0.178; Log-likelihood = −49.99; AIC = 123.98; BIC = 154.87; McFadden R^2^ = 0.235; Hosmer–Lemeshow χ^2^(8) = 2.97, *p* = 0.936. “Unknown” smoking was excluded from modeling. Abbreviations: ACPA, anti-citrullinated protein antibody; AIC, Akaike information criterion; aOR, adjusted odds ratio; AUC, area under the ROC curve; BIC, Bayesian information criterion; CI, confidence interval; COPD, chronic obstructive pulmonary disease; PFT, pulmonary function tests; RA, rheumatoid arthritis; RF, rheumatoid factor.

**Table 4:** Sensitivity analysis: primary model additionally adjusting for pre-index dyspnea and Velcro crackles.

Predictor (Reference)	aOR (95% CI)	*p*
Male sex (ref: female)	5.14 (1.68–15.67)	0.004
Former smoker (ref: never)	0.19 (0.06–0.64)	0.007
Current smoker (ref: never)	2.96 (0.76–11.57)	0.118
Age (per year)	1.04 (0.99–1.09)	0.159
RA duration (per year)	0.96 (0.91–1.01)	0.144
RF positive (ref: negative)	3.02 (0.80–11.42)	0.102
ACPA positive (ref: negative)	0.39 (0.10–1.48)	0.167
COPD/asthma (ref: no)	2.28 (0.78–6.63)	0.130
Ever methotrexate (ref: never)	0.97 (0.20–4.72)	0.972
Ever leflunomide (ref: never)	1.48 (0.56–3.89)	0.429
Current glucocorticoids (ref: no)	2.40 (0.80–7.18)	0.116
Dyspnea (pre-index, yes vs. no)	2.82 (0.84–9.51)	0.094
Velcro crackles (pre-index, yes vs. no)	5.11 (1.32–19.73)	0.018

Model metrics: *N* = 133 (cases = 76, controls = 57); AUC = 0.850; Brier = 0.158; Log-likelihood = −63.91; AIC = 155.83; BIC = 196.40; McFadden R^2^ = 0.301; Hosmer–Lemeshow χ^2^(8) = 12.71, *p* = 0.122. “Unknown” smoking was excluded from modeling. Abbreviations: ACPA, anti-citrullinated protein antibody; AIC, Akaike information criterion; aOR, adjusted odds ratio; AUC, area under the ROC curve; BIC, Bayesian information criterion; CI, confidence interval; COPD, chronic obstructive pulmonary disease; RA, rheumatoid arthritis; RF, rheumatoid factor.

**Table 5:** Sensitivity analysis: conditional logistic regression in matched sample.

Predictor (Reference)	aOR (95% CI)	*p*
RA duration (per year)	0.91 (0.79–1.04)	0.178
RF positive (ref: negative)	2.59 (0.90–24.22)	0.927
ACPA positive (ref: negative)	0.07 (0.00–2.53)	0.146
COPD/asthma (ref: no)	39.12 (1.46–1045.61)	0.029
Ever methotrexate (ref: never)	0.20 (0.02–2.61)	0.220
Ever leflunomide (ref: never)	1.34 (0.22–8.14)	0.749
Current glucocorticoids (ref: no)	3.43 (0.17–70.88)	0.425
Former smoker (ref: never)	0.02 (0.00–1.15)	0.058
Current smoker (ref: never)	1.25 (0.08–19.69)	0.876

Conditional logistic regression stratified by matched set. Matching: greedy 1: up to 2 matching without replacement; sex exact, age ± 5 years, hrCT year ± 2 years. Analysis set after complete-case restriction (and excluding “unknown” smoking): *N* = 77 observations across 29 matched sets (cases = 29, controls = 48). Model metrics: Log-likelihood = −11.80; AIC = 41.59; BIC = 62.69. Abbreviations: ACPA, anti-citrullinated protein antibody; AIC, Akaike information criterion; aOR, adjusted odds ratio; AUC, area under the ROC curve; BIC, Bayesian information criterion; CI, confidence interval; COPD, chronic obstructive pulmonary disease; hrCT, high-resolution computed tomography; RA, rheumatoid arthritis; RF, rheumatoid factor.

## Data Availability

The raw data supporting the conclusions of this article will be made available by the authors on reasonable request.

## Supplementary Materials

The following supporting information can be downloaded at: https://www.mdpi.com/article/10.3390/jcm15072735/s1, Table S1. Shapiro-Wilk normality test for continuous variables; Table S2. Included versus excluded from the primary multivariable model (Table 2); Table S3. Included vs excluded from the matched conditional model (Table 5).

## Abbreviations

The following abbreviations are used in this manuscript:

ACPA	anti–citrullinated protein antibody
AIC	Akaike information criterion
aOR	adjusted odds ratio
AUC	area under the receiver operating characteristic curve
BIC	Bayesian information criterion
b/tsDMARD	biologic/targeted synthetic disease-modifying antirheumatic drug
CI	confidence interval
COPD	chronic obstructive pulmonary disease
CRP	C-reactive protein
DAS28	Disease Activity Score in 28 joints
DLCO	diffusing capacity of the lung for carbon monoxide
EMR	electronic medical record
ESR	erythrocyte sedimentation rate
FEV1	forced expiratory volume in 1 s
FVC	forced vital capacity
HR	hazard ratio
hrCT	high-resolution computed tomography
ILD	interstitial lung disease
NSIP	nonspecific interstitial pneumonia
OR	odds ratio
PFT	pulmonary function test
RA	rheumatoid arthritis
RA-ILD	rheumatoid arthritis-associated interstitial lung disease
RF	rheumatoid factor
ROC	receiver operating characteristic
SD	standard deviation
UIP	usual interstitial pneumonia
ULN	upper limit of normal
