# Supplementary material for: Clinical Factors Associated with hrCT-Confirmed Interstitial Lung Disease in Rheumatoid Arthritis: A Retrospective Case–Control Study
Source: J Clin Med. 2026 Apr 4;15(7):2735. doi: 10.3390/jcm15072735 (PMC13073618; doi:10.3390/jcm15072735)
Supplement: Supplementary file 1 [file jcm-15-02735-s001.zip › jcm-4222212-supplementary.pdf]

Table S1. Shapiro-Wilk normality test for continuous variables

	W Cases	*p*	*W Controls*	*p*
Age, years	0.906	<0.001	0.858	<0.001
RA duration, years	0.837	<0.001	0.828	<0.001
Smoking, pack-years	0.711	<0.001	0.609	<0.001
CRP, mg/L	0.709	<0.001	0.676	<0.001
ESR, mm/h	0.968	0.042	0.897	<0.001
DAS28-CRP	0.947	0.004	0.968	0.141
FVC, % predicted	0.976	0.528	0.992	0.996
FEV1, % predicted	0.989	0.967	0.985	0.881
DLCO, % predicted	0.977	0.500	0.976	0.561

Notes: Shapiro-Wilk tests were performed separately in cases and controls. P-values <0.05 suggest deviation from normality; given multiple tests, these results were used as a descriptive aid rather than for inferential claims.

Abbreviations: CRP C-reactive protein; DAS28 Disease Activity Score in 28 joints; DLCO diffusing capacity of the lung for carbon monoxide; ESR erythrocyte sedimentation rate; FEV1 forced expiratory volume in 1 second; FVC forced vital capacity; RA rheumatoid arthritis.

Table S2. Included versus excluded from the primary multivariable model (Table 2)

	Included (n=134)	*Excluded (n=4)*	*p*	*SMD*
Age, years	66.8 [59.5, 71.6]	66.4 [61.6, 84.5]	0.497	0.639
Female sex	91 (67.9%)	1 (25.0%)	0.108	0.860
Smoking status			0.328	
never	66 (49.3%)	0 (0.0%)		1.143
former	41 (30.6%)	1 (100.0%)		1.458
current	27 (20.1%)	0 (0.0%)		0.669
RA duration, years	8.0 [1.1, 13.8]	0.8 [0.0, 2.9]	0.058	0.975
RF positive	107 (79.9%)	2 (50.0%)	0.194	0.626
ACPA positive	106 (79.1%)	2 (50.0%)	0.206	0.608
CRP, mg/L	9.7 [3.0, 22.7]	8.2 [2.4, 14.3]	0.450	0.569
ESR, mm/h	38.0 [22.2, 61.0]	10.0 [8.5, 14.2]	0.010	1.564
DAS28-CRP	3.8 [2.8, 5.3]	3.4 [3.0, 3.8]	0.430	0.519
COPD/asthma	37 (27.6%)	0 (0.0%)	0.574	0.800
Dyspnea	39 (29.1%)	0 (0.0%)	0.577	0.825
Velcro crackles	35 (26.1%)	0 (0.0%)	0.572	0.775
Any pre-index PFT	97 (72.4%)	2 (50.0%)	0.317	0.459
Ever methotrexate	119 (88.8%)	4 (100.0%)	1.000	0.487
Ever leflunomide	90 (67.2%)	1 (25.0%)	0.114	0.846
Current glucocorticoids	31 (23.1%)	0 (0.0%)	0.575	0.723

Footnotes: Continuous variables are shown as median [Q1, Q3]. P-values are from Mann-Whitney U tests for continuous variables and χ^2/Fisher's exact tests for categorical variables. SMD denotes absolute standardized mean difference. The excluded group is small; therefore, p-values and SMDs should be interpreted cautiously.

Abbreviations: ACPA anti-citrullinated protein antibody; COPD chronic obstructive pulmonary disease; CRP C-reactive protein; DAS28 Disease Activity Score in 28 joints; ESR erythrocyte sedimentation rate; PFT pulmonary function test; RA rheumatoid arthritis; RF rheumatoid factor; SMD Standardized Mean Difference.

Table S3. Included vs excluded from the matched conditional model (Table 5)

	Included (n=77)	*Excluded (n=61)*	*p*	*SMD*
Age, years	66.9 [59.6, 71.6]	65.8 [59.4, 71.2]	0.727	0.066
Female sex	62 (80.5%)	30 (49.2%)	<0.001	0.656
Smoking status			0.104	
never	38 (49.4%)	28 (48.3%)		0.022
former	28 (36.4%)	14 (24.1%)		0.266
current	11 (14.3%)	16 (27.6%)		0.327
RA duration, years	8.0 [1.7, 14.1]	7.0 [1.0, 11.7]	0.250	0.199
RF positive	62 (80.5%)	47 (77.0%)	0.774	0.085
ACPA positive	63 (81.8%)	45 (73.8%)	0.352	0.194
CRP, mg/L	8.6 [2.4, 18.7]	10.2 [3.7, 26.2]	0.292	0.204
ESR, mm/h	33.0 [17.0, 60.0]	44.0 [26.0, 61.0]	0.189	0.149
DAS28-CRP	3.7 [2.8, 5.1]	4.1 [2.9, 5.6]	0.187	0.236
COPD/asthma	23 (29.9%)	14 (23.0%)	0.473	0.157
Dyspnea	21 (27.3%)	18 (29.5%)	0.921	0.050
Velcro crackles	14 (18.2%)	21 (34.4%)	0.048	0.369
Any pre-index PFT	55 (71.4%)	44 (72.1%)	1.000	0.016
Ever methotrexate	69 (89.6%)	54 (88.5%)	1.000	0.035
Ever leflunomide	51 (66.2%)	40 (65.6%)	1.000	0.014
Current glucocorticoids	19 (24.7%)	12 (19.7%)	0.621	0.120

Notes: Continuous variables are shown as median [Q1, Q3]. P-values are from Mann-Whitney U tests for continuous variables and χ^2/Fisher's exact tests for categorical variables. SMD denotes absolute standardized mean difference.

Abbreviations: ACPA anti-citrullinated protein antibody; COPD chronic obstructive pulmonary disease; CRP C-reactive protein; DAS28 Disease Activity Score in 28 joints; ESR erythrocyte sedimentation rate; PFT pulmonary function test; RA rheumatoid arthritis; RF rheumatoid factor; SMD Standardized Mean Difference.
